# Wolfram Syndrome: Diagnosis, Management, and Treatment

**DOI:** 10.1007/s11892-015-0702-6

**Published:** 2016-01-07

**Authors:** Fumihiko Urano

**Affiliations:** Department of Medicine, Division of Endocrinology, Metabolism, and Lipid Research, Washington University School of Medicine, St. Louis, MO 63110 USA; Department of Pathology and Immunology, Washington University School of Medicine, St. Louis, MO 63110 USA

**Keywords:** Wolfram syndrome, Type 1 diabetes, Type 2 diabetes, Blindness, Deafness, Neurodegeneration, β cells, Genetic disorder, Endoplasmic reticulum stress

## Abstract

Wolfram syndrome is a rare genetic disorder characterized by juvenile-onset diabetes mellitus, diabetes insipidus, optic nerve atrophy, hearing loss, and neurodegeneration. Although there are currently no effective treatments that can delay or reverse the progression of Wolfram syndrome, the use of careful clinical monitoring and supportive care can help relieve the suffering of patients and improve their quality of life. The prognosis of this syndrome is currently poor, and many patients die prematurely with severe neurological disabilities, raising the urgency for developing novel treatments for Wolfram syndrome. In this article, we describe natural history and etiology, provide recommendations for diagnosis and clinical management, and introduce new treatments for Wolfram syndrome.

## Introduction

Wolfram syndrome is an autosomal recessive genetic disorder characterized by juvenile-onset diabetes mellitus, diabetes insipidus, optic nerve atrophy, hearing loss, and neurodegeneration. It was first reported in 1938 by Wolfram and Wagener who found four of eight siblings with juvenile diabetes mellitus and optic nerve atrophy [1]. Wolfram syndrome is considered a rare disease and estimated to afflict about 1 in 160,000–770,000 [2, 3]. In 1995, Barrett, Bundey, and Macleod described detailed clinical features of 45 patients with Wolfram syndrome and determined the best available diagnostic criteria for the disease [3]. According to the draft International Classification of Diseases (ICD-11), Wolfram Syndrome is categorized as a rare specified diabetes mellitus (subcategory 5A16.1, Wolfram Syndrome). The prognosis of this syndrome is currently poor as most patients die prematurely with severe neurological disabilities such as bulbar dysfunction and organic brain syndrome, with the median age at death being 30 years (range, 25–49 years), usually from respiratory failure as a result of brain stem atrophy [3, 4]. Although there are currently no effective treatments that can delay, halt, or reverse the progression of Wolfram syndrome, the use of careful clinical monitoring and supportive care can relieve the debilitating symptoms. In this article, we provide recommendations for diagnosis and clinical management and introduce potential new treatments for Wolfram syndrome.

## Common Clinical Presentations and Natural History

**Fig. 1:**
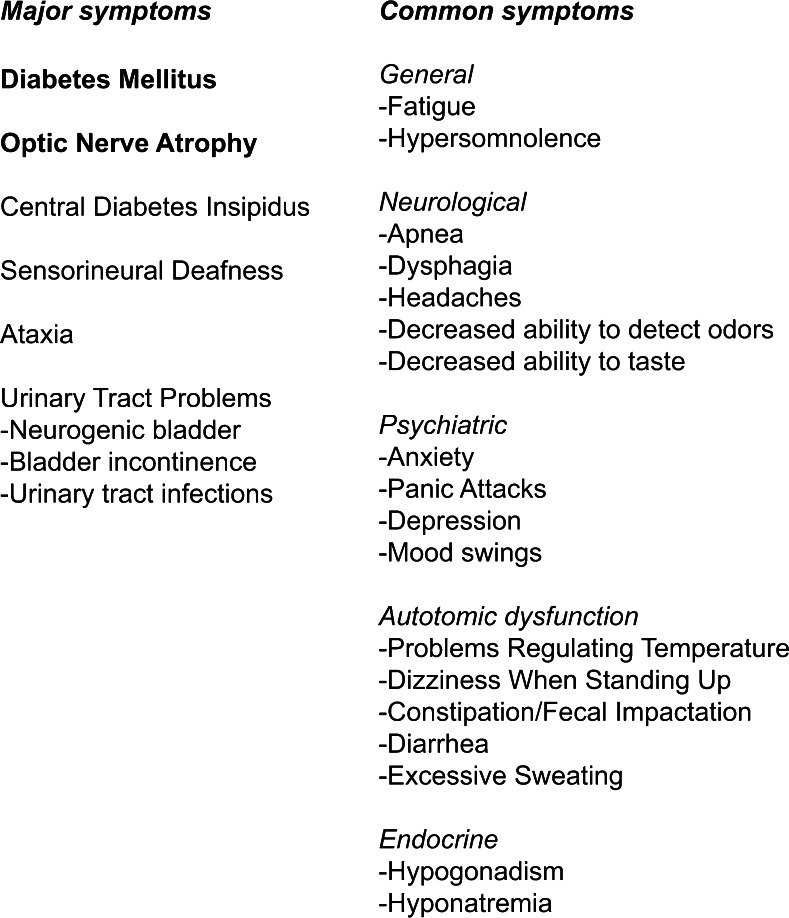
Common clinical manifestations in Wolfram syndrome

The common manifestations of Wolfram syndrome include diabetes mellitus, optic nerve atrophy, central diabetes insipidus, sensorineural deafness, urinary tract problems, and progressive neurologic difficulties. Diabetes mellitus is typically the first manifestation, usually diagnosed around age 6 [3]. Optic nerve atrophy, marked by loss of color vision and peripheral vision, follows around age 11 [3]. Central diabetes insipidus is another common manifestation, affecting approximately 70 % of Wolfram patients [3]. Around 65 % of patients develop sensorineural deafness that can range in severity from deafness beginning at birth to mild hearing loss beginning in adolescence that worsens over time [3, 4]. Urinary tract problems are another major clinical challenge for Wolfram syndrome patients affecting 60 to 90 % of this population. These problems include obstruction of the ducts between the kidneys and bladder, high-capacity atonal bladder, disrupted urination, bladder sphincter dyssynergia, and difficulty controlling urine flow [3]. More than 60 % of patients with Wolfram syndrome develop neurological manifestations, most commonly presenting as problems with balance and coordination (ataxia) beginning in early adulthood. Brain stem atrophy is also a prominent feature that often results in death secondary to central apnea [3, 5, 6]. Other symptoms reported in patients with Wolfram syndrome are listed in Fig. 1 and the Washington University Wolfram Syndrome Clinical Study Website (http://wolframsyndrome.dom.wustl.edu/). Clinical manifestations related to mood disorder and autonomic dysfunction are commonly seen [7, 8]. These are probably related to dysfunction and/or death of neurons due to the defects in the secretion of neurotransmitters and neurotrophic factors. There is currently no effective therapy that can delay the progression of Wolfram syndrome, thus raising the urgency for developing novel treatments.

## Diagnosis of Wolfram Syndrome

### Clinical Diagnosis

It is important for physicians to provide an accurate and prompt diagnosis of Wolfram syndrome, which allows us to provide patients with support and education and initiate appropriate interventions. Suspicion of the diagnosis of Wolfram syndrome is usually based on history and clinical manifestations. Most commonly, the observation of optic nerve atrophy after the diagnosis of diabetes mellitus under the age of 16 triggers the suspicion. Increasing evidence indicates that Wolfram syndrome is a spectrum disorder. Diabetes insipidus, sensorineural deafness, neurological signs including ataxia, autonomic neuropathy, and epilepsy, and neurogenic bladder in combination with diabetes mellitus or optic nerve atrophy could be a sign of Wolfram syndrome. The differential diagnoses include mitochondrial disorders, mutant WFS1 gene-induced deafness, autosomal dominant optic nerve atrophy, Friedreich ataxia, Bardet–Biedl syndrome, and Alstrӧm syndrome.

### Confirmation of the Diagnosis

**Fig. 2:**
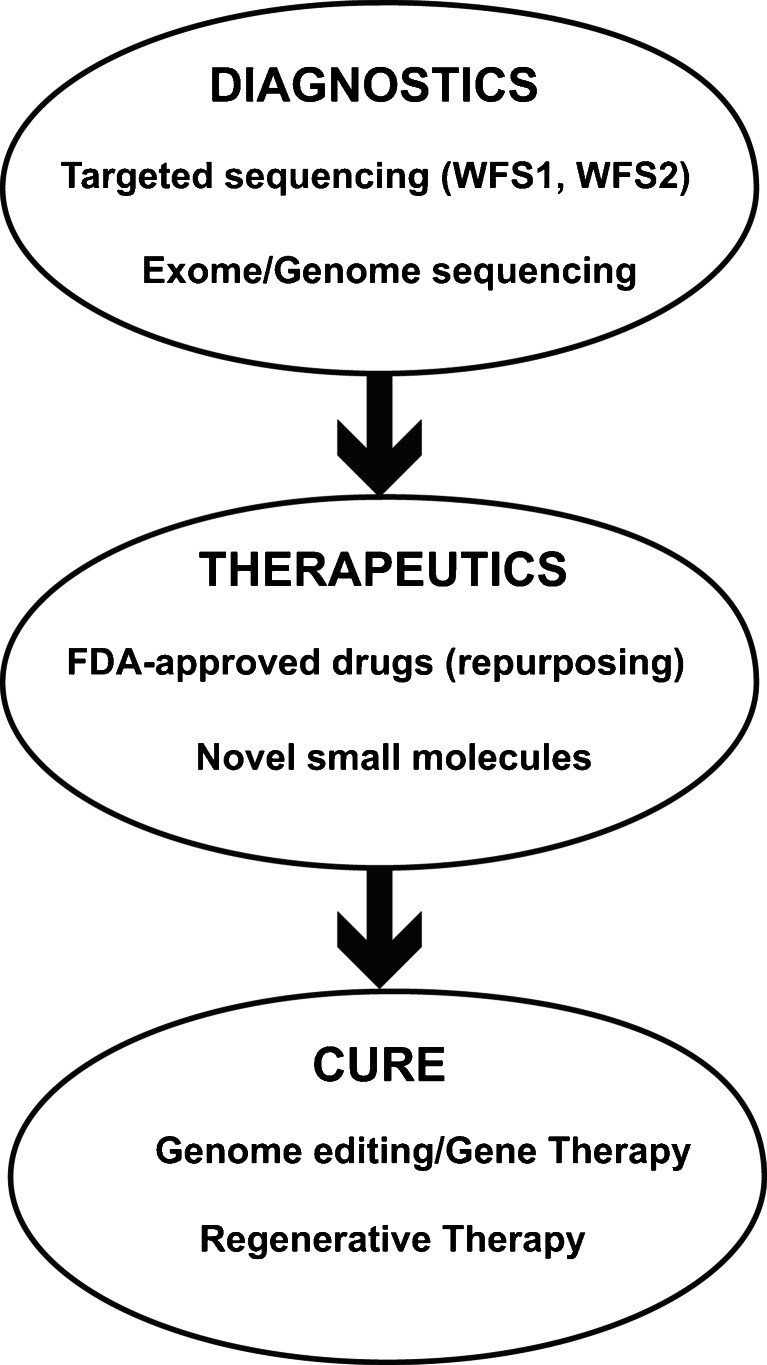
Development of diagnostics and therapeutics for Wolfram syndrome

Although the medical and family histories and the findings of the physical examination are vital for the diagnosis of Wolfram syndrome, genetic testing has been proven to be useful to confirm the diagnosis. The development of genetic tests for this syndrome has identified *WFS1* as the main locus mutated in the majority of this patient population [9]. Sanger sequencing-based genetic testing of the WFS1 gene usually confirms the diagnosis. Majority of patients have recessive mutations in the WFS1 gene, but several dominant mutations have been discovered in our patient population, including H313Y [10]. Dominant mutations in the WFS1 gene are a common cause of low-frequency sensorineural hearing loss [11, 12]. We have recently discovered patients with autosomal dominant diabetes arising from a WFS1 mutation [13]. Thus, the interpretation of genetic testing results requires specialized knowledge. A small number of patients carry recessive mutations in the CISD2 (WFS2) gene [14]. We perform Sanger sequencing-based genetic testing of the WFS2 gene in patients who do not carry mutations in the WFS1 gene. We are currently developing exome sequencing- and genome sequencing-based diagnostic methods for Wolfram syndrome and Wolfram-related disorders (Fig. 2).

## Clinical Management

### Urological Manifestations

Structural and functional urinary tract abnormalities are commonly seen in patients with Wolfram syndrome and significantly affect quality of life. Yearly assessment of renal function, measurement of postvoid residual urine volume by ultrasound, a renal ultrasound, and urodynamic testing are recommended. A large atonic bladder, a low-capacity, high-pressure bladder with sphincteric dyssynergia, and hydroureteronephrosis are common manifestations [3, 4, 15]. We suspect that both bladder dysfunction and upper urinary tract dilatation are primary manifestations although these symptoms may be partially affected by diabetes mellitus and diabetes insipidus. The treatment options for bladder dysfunction include anticholinergic drugs and clean intermittent catheterization. Electrical stimulation and physiotherapy have been effective in some patients.

Recurrent urinary tract infection (UTI) is one of the most common clinical challenges in patients with Wolfram syndrome. The bladder dysfunction caused by the central and peripheral neurologic dysfunction is thought to be the underlying cause of UTI. Urine culture is recommended for Wolfram patients with fever or other symptoms, such as headache. Inflammatory conditions associated with UTI may cause headache and other symptoms.

### Ophthalmologic Manifestations

Optic nerve atrophy with resultant loss of visual acuity is a cardinal feature of Wolfram syndrome and the biggest concern for patients. Yearly eye examination, including visual acuity, color vision testing, fundoscopy, visual field, and optical coherence tomography (OCT) scan, is strongly recommended. Visual evoked potentials are useful to monitor the efficacy of potential treatments. Retinal thinning has been shown to be a reliable marker for the disease progression [16, 17]. Magnification of images on smartphone and tablets, or by magnifying glasses, and voice systems are helpful. Some patients develop cataracts in childhood, and surgery is needed. There are currently no proven effective medical therapies for optic nerve atrophy in Wolfram syndrome. It has been suggested that the use of idebenone or docosahexaenoic acid may be beneficial for delaying the progression of optic nerve atrophy, but the efficacy has not be confirmed by large-scale interventional studies [18, 19••].

### Hearing Manifestations

Sensorineural hearing loss is one of the common symptoms of Wolfram syndrome and observed in around 70 % of patients [20, 21]. The hearing loss affects the high frequencies first and progresses relatively slowly [3, 4, 22]. Routine audiometry is recommended every year or every 2 years. Auditory brainstem response (ABR) audiometry should be performed at diagnosis to confirm the pathophysiology and examine the efficacy of any treatments. Management includes hearing aids and cochlear implant. Hearing manifestations in Wolfram syndrome should be carefully examined because dominant mutations in the WFS1 gene are a common cause of low-frequency sensorineural hearing loss, which is different from Wolfram syndrome [11, 12, 23]. These patients develop low-frequency sensorineural hearing loss but do not develop other symptoms seen in patients with Wolfram syndrome, such as diabetes mellitus and ataxia.

### Neurological Manifestations

Multiple neurological conditions are seen in patients with Wolfram syndrome, including ataxia, central apnea related to brain stem atrophy, autonomic neuropathy, peripheral neuropathy, inability or decreased ability to detect odors, inability or decreased ability to taste, hypersomnolence, headache, and seizures. The following conditions are common major problems in our patients and strongly associated with neurodegeneration with Wolfram syndrome.

#### Ataxia and Dysphagia

Wolfram syndrome is characterized by progressive neurodegeneration. The most common problem is ataxia. Cerebellar ataxia should be assessed yearly or twice a year by neurologists. Dysarthria and swallowing disorders (dysphagia) are commonly seen. Swallowing treatment by a speech-language pathologist is beneficial for patients to prevent aspiration pneumonia. Surgical procedures, including esophageal dilation and esophagomyotomy, have been shown to be effective in some patients. Oral hygiene and dental care are important because dysphagia may lead to the impaired clearance of organisms and pathogenic colonization.

#### Brain Stem Atrophy

Brain stem atrophy is a prominent feature that often results in death secondary to central apnea [3]. Polysomnography, as well as overnight oximetry test, can be used as screening studies. Central apnea should be managed by a pulmonologist. Some patients may require tracheostomy.

#### Autonomic Neuropathy

Autonomic neuropathy is commonly seen in patients with Wolfram syndrome. Signs and symptoms include orthostatic hypotension, anhidrosis, hypohidrosis, hyperhidrosis, constipation, gastroparesis, hypothermia, and hyperpyrexia. A thorough history and review of systems may reveal most of these complaints. Gastrointestinal conditions can be managed by changes in diet, small frequent meals, increased fiber diet, and increased water intake.

#### Headache

Headache may be related to autonomic dysfunction or neuropathy. Some patients experience sharp and stabbing unilateral pain similar to trigeminal neuralgia. A consultation with a neurologist is recommended. Carbamazepine or amitriptyline has been shown to be effective in some patients.

### Psychiatric Manifestations

Anxiety and depression are commonly seen [7, 8, 24]. Psychosis is seen in some patients but not very common. A consultation with a psychiatrist is recommended. Anxiety and depression are treatable in standard way. Cognitive function is usually normal in patients with Wolfram syndrome, especially in younger patients [25].

### Endocrine Manifestations

Diabetes mellitus and diabetes insipidus are the most common clinical manifestations of Wolfram syndrome. These are treatable conditions, and follow-up and management should be done in standard way. The following conditions are associated with endocrine system dysfunction.

#### Hyponatremia

Most patients with Wolfram syndrome have diabetes mellitus and bladder dysfunction in combination with diabetes insipidus. The dose escalation of desmopressin for the treatment of diabetes insipidus should be carefully done because demopressin may cause hyponatremia. Hyponatremia is a common clinical problem in patients with Wolfram syndrome that requires careful management.

#### Hypogonadism

Hypogonadism is seen in some patients. Impaired fertility and erectile dysfunction in male patients and infertility, amenorrhea, and oligomenorrhea in female patients have been reported. These conditions could be treatable and managed in standard way.

## Novel Treatments

### Etiology and Drug Targets

Wolfram syndrome was initially categorized as a mitochondrial disease due to its symptoms and several reports of mitochondrial mutations. However, it has now been established that Wolfram syndrome is a prototype of endoplasmic reticulum (ER) disease [26–28]. The ER is a membrane network within our cells that is involved in protein synthesis, calcium storage, redox regulation, steroid synthesis, cell signaling, and cell death. Previous studies have shown that pancreatic β cells and neurons are particularly sensitive to ER dysfunction, likely due to their high rates of protein synthesis [29]. In Wolfram syndrome, pancreatic β cells and neuronal cells are selectively destroyed as a consequence of mutations in the *WFS1* gene. This gene encodes a transmembrane protein localized to the ER, suggesting that ER dysfunction is a major pathogenic component of Wolfram syndrome. In Wolfram syndrome, *WFS1* mutations lead to elevated ER stress levels, pancreatic β cell dysfunction, and the initiation of ER stress-associated cell death [28, 30]. We have recently reported that depletion of ER calcium and subsequent activation of calpain play a role in β cell death in neurodegeneration in Wolfram syndrome [19••]. A small portion of patients have mutations in the *WFS2* (CISD2) gene [14, 31, 32]. *WFS2* also encodes a transmembrane protein localized to the ER [14]. In patients with *WFS2* mutations, diabetes mellitus and hearing impairment are reported. Their clinical phenotype differs from patients carrying *WFS1* mutations for the absence of diabetes insipidus and for the presence of upper intestinal ulcers and defective platelet aggregation [32], suggesting that there are different and overlapping functions of *WFS1* and *WFS*2.

### Targeting Endoplasmic Reticulum Dysfunction with Currently Available Drugs—Drug Repurposing

One strategy for expediting the development of new therapeutic options for Wolfram syndrome is drug repurposing (Fig. 2) [33]. Drug repurposing is the use of currently approved drugs by regulatory agencies, such as the United States Food and Drug Administration (FDA) and the European Commission and the European Medicines Agency (EMA), to find therapies for other diseases. This is an attractive therapeutic option because the development of novel treatment compounds takes a lot of time and money, which can be avoided by the use of readily available FDA-approved drugs. In the treatment of Wolfram syndrome, drugs can be targeted to several areas of ER dysfunction including modulators of ER stress, ER calcium homeostasis, and cellular proteostasis [34–36].

Chemical chaperones are small compounds which can stabilize protein conformation during folding and improve trafficking of mutant proteins through the ER [37]. Currently, there are two FDA-approved chemical chaperones, 4-phenylbutyric acid (PBA) and tauroursodeoxycholic acid (TUDCA). In WFS1-deficient β cells, there is an increased level of ER stress and decreased insulin content, both of which can be normalized by treatment with chaperones [38•]. This suggests that PBA and TUDCA may improve β cell functions and prevent ER stress-mediated β cell death and neurodegeneration in patients with Wolfram syndrome.

Proper function of the ER requires a steep calcium gradient; without this gradient, cells undergo ER stress and cell death. Thus, targeting drugs known to maintain ER calcium levels during ER stress presents a novel therapeutic target for Wolfram syndrome. In β cells, ER stress has been shown to elicit ER calcium depletion resulting in β cell death, which can be prevented with pharmacologic restoration of ER calcium levels [39]. Several currently FDA-approved compounds have been shown to preserve ER calcium levels in response to ER stress. We have recently discovered that dantrolene which targets ryanodine receptor localized to the ER membrane prevents β cell death and neurodegeneration in animal models and cell models with Wolfram syndrome [19••]. Interventional studies using dantrolene and other ER calcium stabilizers should be considered.

### Development of Drugs Targeting the Endoplasmic Reticulum

Because currently available drugs are not specifically designed to target ER stress or ER dysfunction, we should create novel small molecules to control ER homeostasis in Wolfram syndrome in addition to using FDA-approved drugs (Fig. 2). There are three major pillars that sustain ER homeostasis: calcium homeostasis, redox regulation, and protein folding. Accumulating evidence suggests that WFS1 protein plays a role in maintaining ER calcium homeostasis, raising the possibility that small molecules that can regulate ER calcium levels may prevent cell death in Wolfram syndrome. One of the major genes required for ER calcium homeostasis in β cells is sarco/endoplasmic reticulum Ca^2+^-ATPase (SERCA) [40]. It has been reported that WFS1 binds to SERCA and modulates its function [41]. Thus, a small molecule that can activate SERCA and maintain high ER calcium levels under pathological conditions could prevent death of neurons and β cells in Wolfram syndrome. Another possibility is to target calcium channels in the ER, such as ryanodine receptors and inositol trisphosphate receptors. We have recently reported that dantrolene which targets ryanodine receptors can prevent the death of neurons and β cells in Wolfram syndrome using mouse models and induced pluripotent stem cell (iPSC) models of the disease [19••]. We are currently developing novel therapeutics using small molecules targeting these mediators of ER calcium homeostasis in collaboration with the drug development team at the National Center for Advancing Translational Sciences, National Institutes of Health.

### Regenerative Therapy and Gene Therapy for Wolfram syndrome

Our first goal is to stop the progression of Wolfram syndrome using FDA-approved drugs or novel small molecules targeting the ER. In parallel, we need to figure out a way to replace damaged tissues, such as pancreatic β cells and retinal cells, in patients with Wolfram syndrome. Rapid progress in the field of regenerative medicine may make this possible in the near future [42] (Fig. 2). Our strategy is to create induced pluripotent stem (iPS) cells using patients’ skin cells, correct *WFS1* gene mutations with genome editing technology, and differentiate these iPS cells into insulin-producing β cells, retinal cells, and neurons for transplantation [43•]. We are currently using the Clustered Regularly Interspaced Short Palindromic Repeats (CRISPR) technology. We are also testing if mesencephalic astrocyte-derived neurotrophic factor (MANF), a regeneration factor purified from astrocytes, can prevent cell death and activate the proliferation of remaining β cells, neurons, and retinal ganglion cells by leveraging our natural ability to regenerate damaged tissues [44, 45].

## Conclusion

### Multidisciplinary Care and Partnership with Patient Organizations

Wolfram syndrome affects different organs and systems in the body. Thus, multidisciplinary care by physicians and healthcare professionals from a range of disciplines is required. Based on our experience, a strong patient-doctor partnership can facilitate the communication between physicians in different specialties. Patient organizations have played an important role to encourage physicians from different specialties, clinics, hospitals, and even countries to work together. The strong partnership between physicians, researchers, and patient organizations is also important to conduct successful clinical trials.

### Power of Wolfram Syndrome

Increasing evidence indicates that ER stress and ER dysfunction play important roles in the pathogenesis of common diseases, such as type 1 and type 2 diabetes, as well as multiple neurodegenerative diseases [46]. Its monogenic etiology makes Wolfram syndrome more amenable to revealing the mechanisms of ER stress-mediated cell death than other common conditions in which multiple factors typically interact to produce the disease manifestations. Thus, Wolfram syndrome represents an ideal model to shed new light on the underlying causes of β cell death in diabetes, neurodegeneration, and retinal cell death mediated by ER dysfunction.

**Fig. 3:**
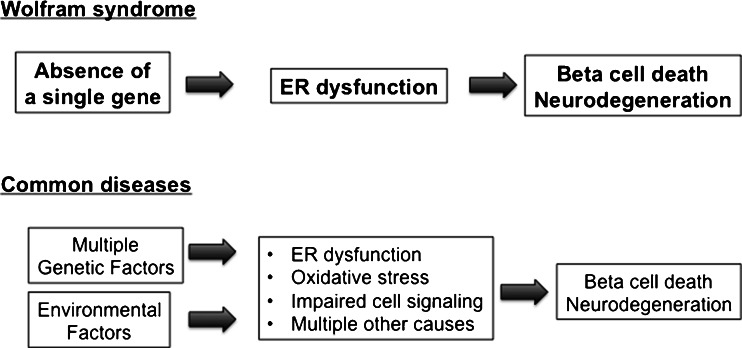
Power of Wolfram syndrome. It has been established that endoplasmic reticulum (ER) dysfunction and ER stress are critical pathogenic components of Wolfram syndrome. It would be possible to identify biomarkers and treatments targeting ER in mechanistically homogenous Wolfram syndrome patients, which may lead to a breakthrough for treatments of common diseases, such as type 1 diabetes, type 2 diabetes, and neurodegeneration, in which ER dysfunction is involved

We believe in the strong power of Wolfram syndrome to understand the pathogenesis and develop novel therapeutic modalities for more prevalent diseases. Our study on Wolfram may lead to a breakthrough for treatments of not only Wolfram syndrome but also common diseases, such as type 1 diabetes, type 2 diabetes, and neurodegeneration, in which ER dysfunction is involved (Fig. 3).
